# Exploring Factors That Predict Marketing of e-Cigarette Products on Twitter: Infodemiology Approach Using Time Series

**DOI:** 10.2196/37412

**Published:** 2022-07-22

**Authors:** Nnamdi C Ezike, Allison Ames Boykin, Page D Dobbs, Huy Mai, Brian A Primack

**Affiliations:** 1 College of Education and Health Professions University of Arkansas Fayetteville, AR United States; 2 College of Engineering University of Arkansas Fayetteville, AR United States; 3 College of Public Health and Human Sciences Oregon State University Corvallis, OR United States

**Keywords:** tobacco, electronic cigarettes, social media, marketing, time series, youth, young adults, infodemiology, infoveillance, digital marketing, advertising, Twitter, promote, e-cigarette

## Abstract

**Background:**

Electronic nicotine delivery systems (known as electronic cigarettes or e-cigarettes) increase risk for adverse health outcomes among naïve tobacco users, particularly youth and young adults. This vulnerable population is also at risk for exposed brand marketing and advertisement of e-cigarettes on social media. Understanding predictors of how e-cigarette manufacturers conduct social media advertising and marketing could benefit public health approaches to addressing e-cigarette use.

**Objective:**

This study documents factors that predict changes in daily frequency of commercial tweets about e-cigarettes using time series modeling techniques.

**Methods:**

We analyzed data on the daily frequency of commercial tweets about e-cigarettes collected between January 1, 2017, and December 31, 2020. We fit the data to an autoregressive integrated moving average (ARIMA) model and unobserved components model (UCM). Four measures assessed model prediction accuracy. Predictors in the UCM include days with events related to the US Food and Drug Administration (FDA), non-FDA-related events with significant importance such as academic or news announcements, weekday versus weekend, and the period when JUUL maintained an active Twitter account (ie, actively tweeting from their corporate Twitter account) versus when JUUL stopped tweeting.

**Results:**

When the 2 statistical models were fit to the data, the results indicate that the UCM was the best modeling technique for our data. All 4 predictors included in the UCM were significant predictors of the daily frequency of commercial tweets about e-cigarettes. On average, brand advertisement and marketing of e-cigarettes on Twitter was higher by more than 150 advertisements on days with FDA-related events compared to days without FDA events. Similarly, more than 40 commercial tweets about e-cigarettes were, on average, recorded on days with important non-FDA events compared to days without such events. We also found that there were more commercial tweets about e-cigarettes on weekdays than on weekends and more commercial tweets when JUUL maintained an active Twitter account.

**Conclusions:**

e-Cigarette companies promote their products on Twitter. Commercial tweets were significantly more likely to be posted on days with important FDA announcements, which may alter the narrative about information shared by the FDA. There remains a need for regulation of digital marketing of e-cigarette products in the United States.

## Introduction

Use of electronic nicotine delivery systems (known as electronic cigarettes, vapes, or e-cigarettes) has increased substantially over the past decade, particularly among young populations (youth, those aged under 18 years, and young adults, those aged 18-24 years) [1,2]. E-cigarettes use among these young populations is particularly concerning due to the risks of cardiovascular and respiratory illnesses that these devices can have for those who would not otherwise use tobacco products [3-5]. Further, the addiction potential of these novel tobacco products, especially newer models that contain excessive levels of nicotine, has caused many in the public health community to question if this new technology could create a new generation of smokers, reversing declines in smoking rates and hard-fought public health milestones [6,7].

Recent data suggest that e-cigarette use is most common among those aged 18 to 44 years [2]. People in these age groups are the most active users of Twitter, one of the most popular social media platforms [8]. As of April 2021, 76% of Twitter’s 300 million active users were aged 18 to 49 years. With a maximum of 280-character length, messages containing personal information or views about products such as e-cigarettes can be shared by users. Users’ posts on Twitter are referred to as tweets.

Emery and colleagues [9] suggest that, when compared to non–e-cigarette users, users of e-cigarette products were more likely to be exposed to information about e-cigarettes via social media platforms, such as Twitter and Facebook, and other mediums like television content, email, and the internet. e-Cigarette content to which social media users are exposed includes tobacco marketing and promotional material [10-12]. This type of advertising on social media helps tobacco companies target users based on their demographic information [13,14]. However, although there has been significant work around the content analysis of commercial tweets about e-cigarettes on social media [15-17], little is known about the factors that drive how often manufacturers of e-cigarettes promote their products on social media.

In 1971, the US Congress outlawed tobacco advertisements on radio and television. Since that time, manufacturers of tobacco products have sought alternative ways to market their products, including marketing campaigns on the internet and social media. Digital marketing, currently unregulated in the United States, offers tobacco (and e-cigarette) companies the opportunity to reach a wide audience [10,18]. This includes social media platforms such as Facebook, Twitter, YouTube, and TikTok [11,19,20]. For example, Huang and colleagues [20] examined the marketing of e-cigarettes on Twitter and found 89.6% of e-cigarette tweets to be commercial tweets. Similarly, Kim and colleagues [11] identified 1.7 million tweets about e-cigarettes spanning over 5 years and found that 93.4% of these tweets advertised e-cigarettes. Social media, therefore, provides a largely unguarded platform for marketing e-cigarette products that has important public health implications.

Social media marketing of e-cigarette products may come from individual accounts, paid corporate advertisements, and paid corporate “influencers” [21]. For example, Jackler and colleagues [22] noted that JUUL, a major e-cigarette company, paid influencers (private social media users with large numbers of followers) to “increase brand awareness and inspire sales.” This type of marketing has been associated with the use of e-cigarettes, especially among adolescent audiences [23]. Social media platforms such as Facebook, Instagram, and Twitter prohibit advertisement of tobacco products [24,25]. This restriction only applies to paid advertising. This means that tobacco companies may still market their products on social media via posts and tweets but cannot use paid advertising, which can be specifically used to target users of certain demographic groups.

Although e-cigarette advertisements are currently not regulated, the US Food and Drug Administration (FDA) has the authority to regulate tobacco products in the United States, including manufacture, distribution, and marketing. On March 17, 2021, the FDA requested that 4 e-cigarette companies disclose information about their marketing practices [26]. Part of the request included information on social media advertising and marketing plans, as well as plans to target specific audiences. Given the FDA’s limitations on exploring each e-cigarette company’s social media marketing, research is needed to understand the factors that predict how tobacco companies conduct brand marketing of their products on social media. Kim and colleagues [11] described the features of commercial tweets about e-cigarettes, including the type of products contained in the advertisement, the number of active accounts, and the type of advertising (promotion, coupon, percent off, and discount). Although these features capture the characteristics of the commercial tweets, they contain little information about the factors that trigger these commercial companies to aggressively promote their products. Thus, the purpose of this study was to determine the best approach for modeling commercial Twitter data on marketed e-cigarette products. This study also sought to explore factors associated with commercial Twitter marketing of e-cigarette products.

## Methods

### Data Collection and Annotation

The data analyzed in this study are tweets about e-cigarettes between January 1, 2017, and December 31, 2020. The tweets were collected daily using the real-time infoveillance of Twitter health messages (RITHM) open-source software [27]. Using the Twitter streaming application programming interface, the RITHM software gathers key information about each tweet, including the number of duplicate tweets based on the tweet ID, where the software automatically saves duplicate tweets as 1 single tweet record. This was crucial to our analysis as it prevented the factor of tweets or retweets with the same text from influencing our findings. We used search terms that capture Twitter chatter related to e-cigarettes, similar to past research [28-30], including words such as vape, vapes, vaper, vapers, vaping, JUUL, JUULs, JUULing, and tobacco. A total of 1% (n=2401) of the tweets posted between August 23, 2019, and September 25, 2019, were selected for annotation by 2 independent researchers. The date range was selected based on a particularly high volume of tweets posted for the given dates. Further, selected tweets were stratified by day to account for volume changes in the number of tweets and to accurately represent Twitter discussions over time. Previous work [27,31,32] established that this sample size and selection method provided adequate representation of tweets made within the selected time frame.

The procedures developed by Crabtree and Miller [33] for public health qualitative research served as a guide for developing the codebook used for human annotation. The first step involved an inductive procedure [34]. Using in vivo coding, 3 researchers explored 200 tweets searching for nuanced information related to e-cigarette–related tweets. Next, the team refined the codebook by adding, splitting, expanding, or deleting codes, an inductive procedure used during qualitative data analyses [34,35]. Relevant tweets were coded as dichotomous indicators, denoting whether the tweet referred to vaping in the context of e-cigarettes. For example, the following tweet was classified as a relevant tweet: “Omg!!!!! Mine is getting interrupted by a vaping special. Coming on at 11pm here. _emoj_weary_ _emoj_weary_ _emoj_weary_ I am tired.” If the tweet did not mention e-cigarettes or referred to vapor in an unrelated context, it was removed from further analysis. Subsequently, we identified promotional posts about tobacco products that appeared to be advertisements or marketing for vaping products. These posts were classified as commercial tweets. For example, the following tweet was classified as a commercial tweet: “COCO THC CBD Oil # Vape System New pod Style THC # CBD Oil System 4 empty tanks that are easy to fill and a 220ohm slim battery. Share !”

Two coders were provided with online versions of the 2401 tweets for annotation using a qualitative content analysis approach. Coders were also provided with retweets, which are tweets that are in response to other users’ tweets. Coding 500 tweets each week, annotators classified tweets as commercial if the tweets were commercial promotion of e-cigarettes and noncommercial if otherwise. Cohen kappa [36] measure of interrater agreement reveal a high coder agreement (*κ*>.80) on classification of relevant and commercial tweets, indicating over 80% agreement between coders after accounting for chance agreement.

### Classification of Tweets

Tweets annotated by human coders were used to train a model to classify the remaining tweets. In this study, classification was performed using a classifier that was pretrained and fine-tuned on BERTweet, a variation of Google AI Language’s bidirectional encoder representations from transformers (BERT). Pretrained on English tweets, BERTweet improves on other transformer models used for natural language processing tasks by enhancing the transformer’s capability of recognizing important words in a given text sequence [37]. This is accomplished by the masking and next sentence prediction objectives performed in the pretraining layers of BERTweet [38], along with the pretraining optimizations of the “robustly optimized BERT pretraining approach” to address the significant undertraining of BERT [39]. As the model uses the encoder representation of a transformer, BERTweet can be fine-tuned for classification tasks.

### Ethics Approval

This study did not use human participants. Data were collected from publicly available platforms and require no ethics approval.

### Modeling Techniques

One of the goals of this study was to find the best approach for modeling time series data to predict commercial Twitter activities about e-cigarettes and vaping. Time series models can provide tools to predict or forecast future events based on past trends. Time series modeling has been extensively used in public health research to predict coronavirus disease spread, study Zika epidemic case counts, and understand changes in public health opinions due to coronavirus restrictions [40-42]. This study compared the performance of the autoregressive integrated moving average (ARIMA) [43] model and unobserved components model (UCM) [44] in predicting commercial Twitter activities about e-cigarettes and vaping.

#### ARIMA Approach

The ARIMA model can be expressed as

yt = ϕ0 + ϕ1yt–1 + ... + ϕpyt–p + θ1εt–1 + ... + θqεt–q + ε*t* (1)

where *t* is the time point, *y_t_* is the forecast variable which is the frequency of commercial tweets at time *t*, *ϕ_i_* is the coefficient for the autoregressive term *p*, *θ_j_* is the coefficient for the moving average term *q*, and *ε_t_* is the random error at time *t*. The ARIMA modeling technique consists of 3 steps: model identification, parameter estimation, and model diagnostic checking. These steps were performed to optimize the ARIMA model for assessing the frequency of commercial tweets. First, the amount of differencing and the lag size were determined at the model identification stage. ARIMA models are based on the assumption of stationarity of the differenced series [45]. Second, we verified that the stationarity and homoscedasticity assumptions were satisfied after model estimation. Third, diagnostic plots such as autocorrelation function (ACF) and partial autocorrelation function (PACF) plots were examined to assess if the fitted models were appropriate. The ACF plot provides the correlation between observations at time *t* and at time *t–k* (where *k* is the number of lags). It is preferred to have autocorrelations near zero for all lags. The PACF plot provides the correlation between observations at time *t* and the residuals at previous lags. Essentially, PACF removes the components that have been explained by previous lags. The PACF plot is a useful tool for determining the order of the autoregressive term. Finally, we selected the appropriate autoregressive (AR) parameter (*p*) and moving average (MA) parameter (*q*) based on the ACF and PACF plots.

#### UCM Approach

One of the main advantages of the UCM approach over the ARIMA approach is that researchers can identify and introduce additional explanatory variables. The explanatory variables could be intervention variables that are useful in explaining patterns in the series [44]. In addition, UCM is efficient in handling missing observations [45]. In the UCM modeling framework, the series is decomposed into trend, seasonal, cyclical, and autoregressive components. In addition, the UCM models regression effects due to the predictor series. The UCM can be expressed as



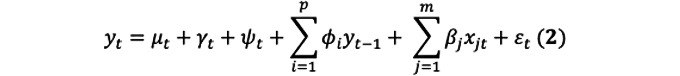



where *t* is the time point, *y_t_* is the forecast variable which is the frequency of commercial tweets at time *t*, *μ_t_* is the trend component, *γ_t_* is the seasonal component, and *ψ_t_* is the cyclical component. The term 
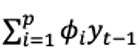
 is used to model the autoregressive regression component based on past observations of the series. The term 
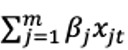
 captures explanatory regression predictors where *x_jt_* is the observed value of predictor *x_j_* at time *t* and *β_j_* is the regression slope for predictor *x_j_*. Finally, *ε_t_* is a white noise error term.

We included 4 explanatory variables in the UCM used in this study: (1) FDA-related events, (2) other (non-FDA) events, (3) day of the week, and (4) JUUL.

##### FDA Variable

Drug Watch International and Consumer Advocates for Smoke-Free Alternatives Association (CASAA) maintain a timeline of events of vaping and e-cigarettes. We reviewed the timeline to identify days with FDA-related events such as announcements about vaping/e-cigarettes, campaigns, and court rulings. The FDA variable was dummy coded. Days in which there were FDA-related events were coded as 1 and 0 if otherwise.

##### Other Variable

The Drug Watch and CASAA timeline of events was also used to create a variable for other events. These events were events of high importance that were non–FDA-related. For example, other events included state legislative actions controlling the use of e-cigarette products and significant scientific research studies reported in national news. The variable on other events about e-cigarettes was also dummy coded. A value of 1 was used to indicate a day with such an event.

##### JUUL Variable

We also included a variable referred to as JUUL in the model. We included this variable in order to understand the impact of JUUL’s tweet activities on the frequency of commercial tweets about e-cigarettes. JUUL is the most popular e-cigarette brand accounting for 76% of e-cigarette retail sales [46]. JUUL has a corporate Twitter page. Of note, JUUL stopped tweeting from its corporate Twitter account on August 29, 2019. We included a dummy coded variable by assigning a value of 1 to indicate periods that JUUL was tweeting and 0 for the period when they stopped tweeting (ie, after August 29, 2019). We will refer to the periods when JUUL was tweeting as “active” and the periods of prolonged inactivity as “inactive.”

##### Day Variable

Finally, a dummy-coded day variable was included in the model to indicate whether the commercial tweet was promoted on a weekend (value of 1) or weekday (value of 0).

### Data Analysis

All analyses were performed in SAS (version 9.4, SAS Institute Inc). There were 1401 out of 1460 days with complete data. A RITHM software outage resulted in failure to collect 59 days of data. Missing observations may bias the forecasting ability of time series models. Jalles [45] noted that it is difficult to the use ARIMA model in the presence of missing data. However, the UCM procedure handles missing values efficiently and can be extended to ARIMA models [47,48]. Both the ARIMA model and UCM were fitted using the UCM procedure in SAS [48].

We took an iterative modeling approach to determine the best fitting UCM. First, we specified a UCM with trend and irregular components. Next, we examined the parameter estimates of the components to determine whether to treat them as stochastic or deterministic. Nonsignificant (deterministic) components were removed from the model. Finally, the 4 explanatory variables used in this study were included in the model (ie, day, FDA event, non-FDA event, and JUUL). At each step, the ACF and PACF plots served as diagnostic tools for assessing the fitted models.

### Model Evaluation

The performance of our models was evaluated using root mean square error (RMSE), mean absolute percentage error (MAPE), mean absolute deviation (MAD), and coefficient of determination (*R*^2^).

#### Root Mean Square Error

RMSE gives the overall measure of accuracy of how well the model predicts the frequency of daily commercial tweets. The RMSE for each model was computed using



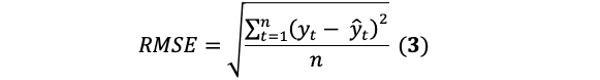



where *y_t_* is the frequency of commercial tweets at time *t*, 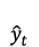 is the predicted frequency of commercial tweets at time *t* based on the fitted model, and *n* is the number of observations.

#### Mean Absolute Percentage Error

MAPE measures the accuracy of the model in terms of percentage error. The MAPE for each model was computed using



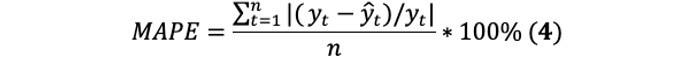



where *y_t_* is the frequency of commercial tweets at time *t*, 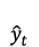 is the predicted frequency of commercial tweets at time *t* based on the fitted model, and *n* is the number of observations. Smaller values of the MAPE indicate fewer prediction errors, hence the best fitting model will have a smaller MAPE.

#### Mean Absolute Deviation

MAD is the average of the absolute value of the deviation between the observed frequency of commercial tweets and the predicted frequency of commercial tweets based on the fitted model. Essentially, MAD provides the amount of prediction errors in the same units as the observed counts. The MAD for each model was computed using



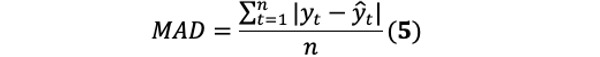



where *y_t_* is the frequency of commercial tweets at time *t*, 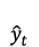 is the predicted frequency of commercial tweets at time *t* based on the fitted model, and *n* is the number of observations. Smaller values of the MAD are preferred.

#### Coefficient of Determination

The *R*^2^ (coefficient of determination) statistic measures the proportion of variance in the frequency of commercial tweets which is accounted for by the predictors. The *R*^2^ statistic is computed as



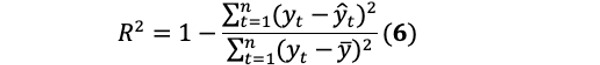



where *y_t_* is the frequency of commercial tweets at time *t*, 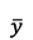 is the average frequency of commercial tweets, 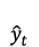 is the predicted frequency of commercial tweets at time *t* based on the fitted model, and *n* is the number of observations. A larger *R*^2^ statistic is preferred.

## Results

### Tweet Classification Results

#### Classifier Settings

**Figure 1:**
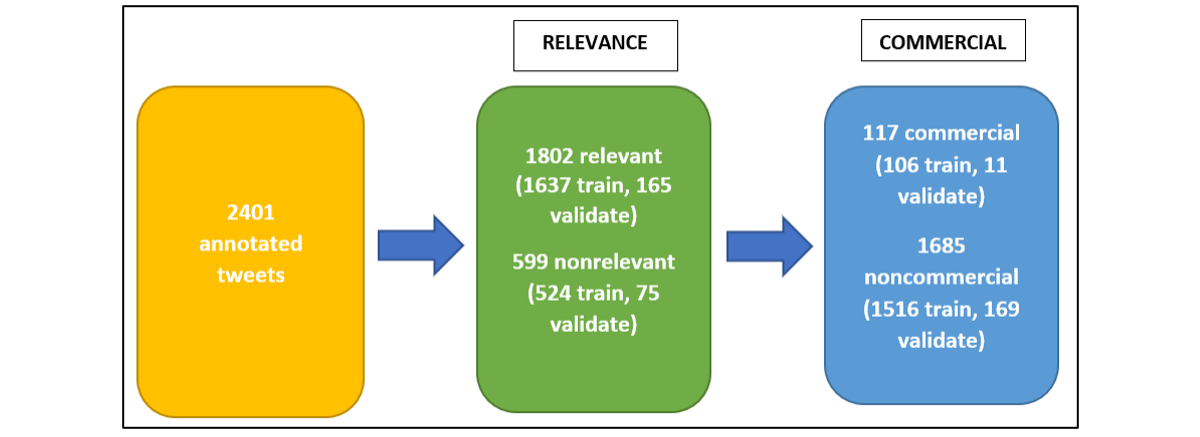
Filtering process of the 2401 tweets used to train and validate the BERTweet classifiers.

Two BERTweet classifiers were trained using the set of annotated tweets: one for relevance and another for commercial. The number of tweets used to train and validate each classifier is provided in Figure 1. The sets of tweets for relevance and commercial were each split randomly to where 90% of the tweets were used to train and fine-tune the model while the remaining 10% was used to validate the model. For the hyperparameters, each BERTweet classifier was trained for 20 epochs with a batch size of 32 and learning rate of 5×10^–5^. For comparison, we used the long short-term memory (LSTM) model proposed by Visweswaran et al [28], which was trained for 5 epochs and a batch size of 64 under the same splits on the annotated data set as the BERTweet classifiers. As part of a previous study analyzing the trend in the commercial nature of tweets related to vaping, this LSTM model was found to have the highest classification accuracy when tested against other deep learning classifiers such as convolutional neural network (CNN), LSTM-CNN, and bidirectional LSTM [28].

#### Classifier Results

We measured the performance of the classifiers using *F*_1_, which is a function of precision and recall, and area under the receiver operating characteristic (AUROC), which measures the discrimination of the classifiers. For the task of classifying a tweet as relevant or nonrelevant, the BERTweet classifier obtained an *F*_1_-score of 0.976 and an AUROC score of 0.945 while the LSTM model had an *F*_1_-score of 0.924 and an AUROC score of 0.924. In classifying tweets as commercial or noncommercial, the BERTweet classifier produced an *F*_1_-score of 0.990 and an AUROC score of 0.993. In comparison, the LSTM classifier achieved an *F*_1_-score of 0.727 and an AUROC score of 0.903.

### Descriptive Statistics

**Figure 2:**
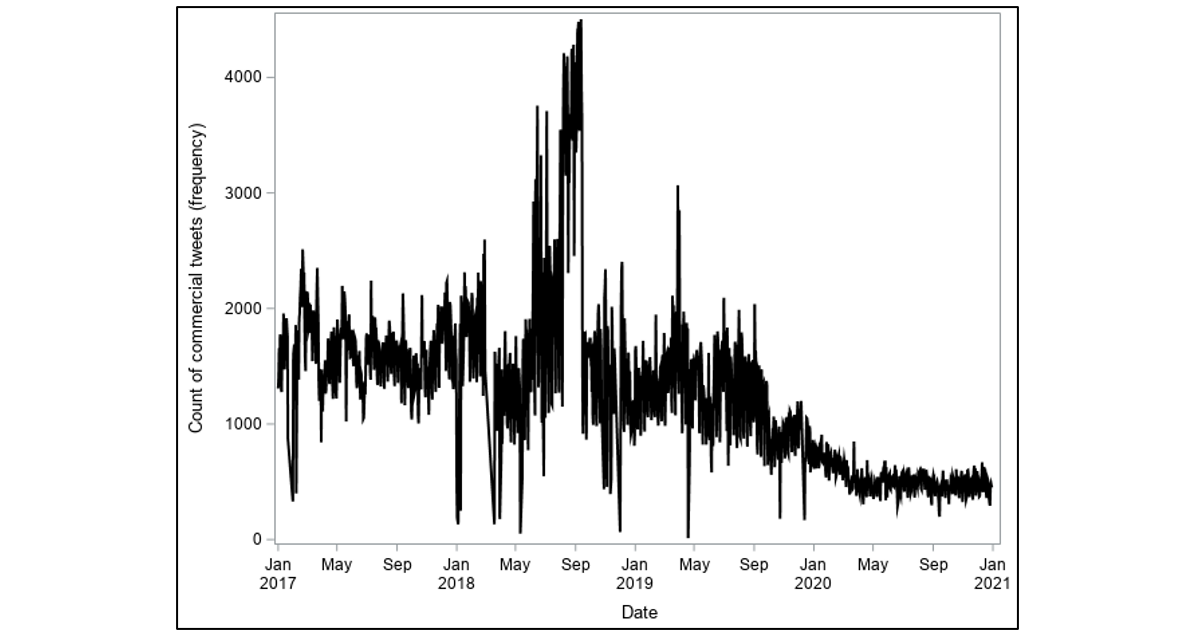
Daily frequency of commercial tweets from January 1, 2017, to December 31, 2020.

**Figure 3:**
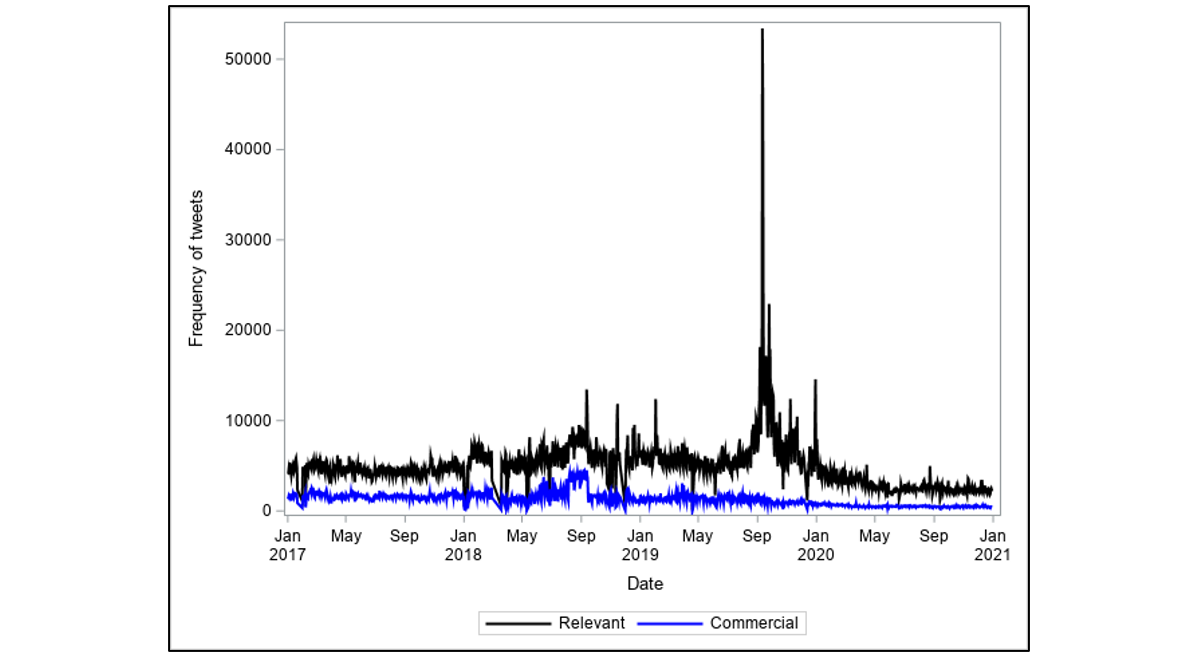
Daily frequency of relevant and commercial tweets from January 1, 2017, to December 31, 2020.

A total of 1,821,603 commercial e-cigarette tweets were recorded from January 1, 2017, to December 31, 2020. Figure 2 presents the daily frequency of commercial tweets. On average, there were 1300 commercial tweets per day, and the frequency of tweets was highly variable with a standard deviation of 718. Figure 3 presents a visual comparison of the daily frequency of relevant (ie, tweets that referred to vaping in the context of e-cigarettes) and commercial tweets about e-cigarettes. On average, 26% (SD 9.3%) of the relevant tweets were brand marketing of e-cigarette products. Brand marketing of e-cigarettes on Twitter declined over the 4-year period. In 2017, the average percentage of commercial tweets was 35% (SD 3.5%). This dropped to an average of 30% (SD 8.9%) in 2018 and an average of 20% (SD 7.3%) in 2019. Finally, following Twitter’s ban on paid advertising, only 19% (SD 3.2%) of the relevant tweets in 2020 were classified as commercial tweets.

Table 1 presents the descriptive statistics of the explanatory variables investigated. On average, the mean frequency of daily commercial tweets on days with FDA-related events was 1447.60 (SD 659.08) compared to 1295.10 (SD 719.61) on days without FDA events. Similarly, on average, there were more commercial tweets on days with other non-FDA events (mean 1336.21, SD 604.61) and on weekdays (mean 1390.20, SD 585.85). The average number of daily commercial tweets when JUUL maintained an active account was over 1000 tweets higher than when JUUL stopped tweeting from its corporate account.

**Table 1:** Summary of daily frequency of commercial tweets for each predictor.

Predictor and description of level		Values, mean (SD)
**FDA^a^**		
	FDA event (n=47)	1447.60 (659.08)
	No FDA event (n=1354)	1295.10 (719.61)
**Other**		
	Other event (n=137)	1336.21 (604.61)
	No other event (n=1264)	1296.31 (729.30)
**JUUL**		
	Active^b^ account (n=920)	1648.76 (630.19)
	Inactive account (n=481)	633.56 (254.82)
**Day**		
	Weekend (n=395)	1071.04 (744.84)
	Weekday (n=1006)	1390.20 (585.85)

^a^FDA: US Food and Drug Administration.

^b^Active is defined as periods when JUUL was tweeting from its corporate Twitter account.

### Model Estimation Summary

#### ARIMA Approach

**Figure 4:**
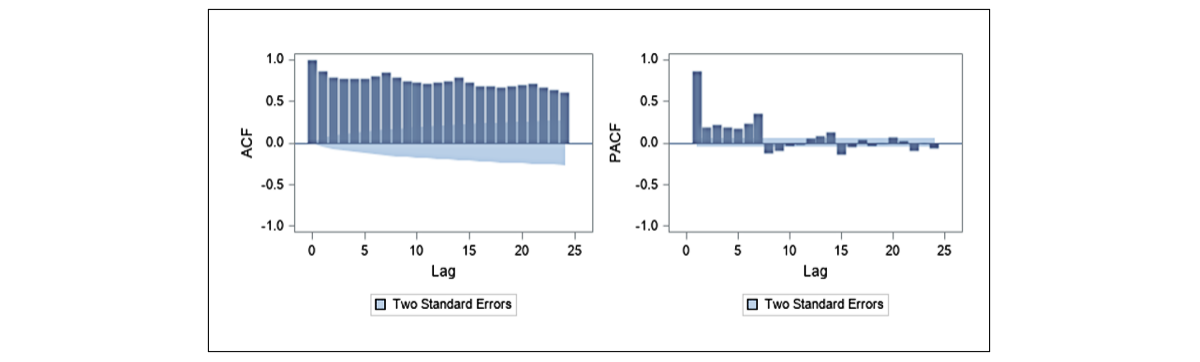
Autocorrelation function (left panel) and partial autocorrelation function (right panel) plots for daily commercial tweets about e-cigarettes before differencing for the autoregressive integrated moving average model. ACF: autocorrelation function; PACF: partial autocorrelation function.

**Figure 5:**
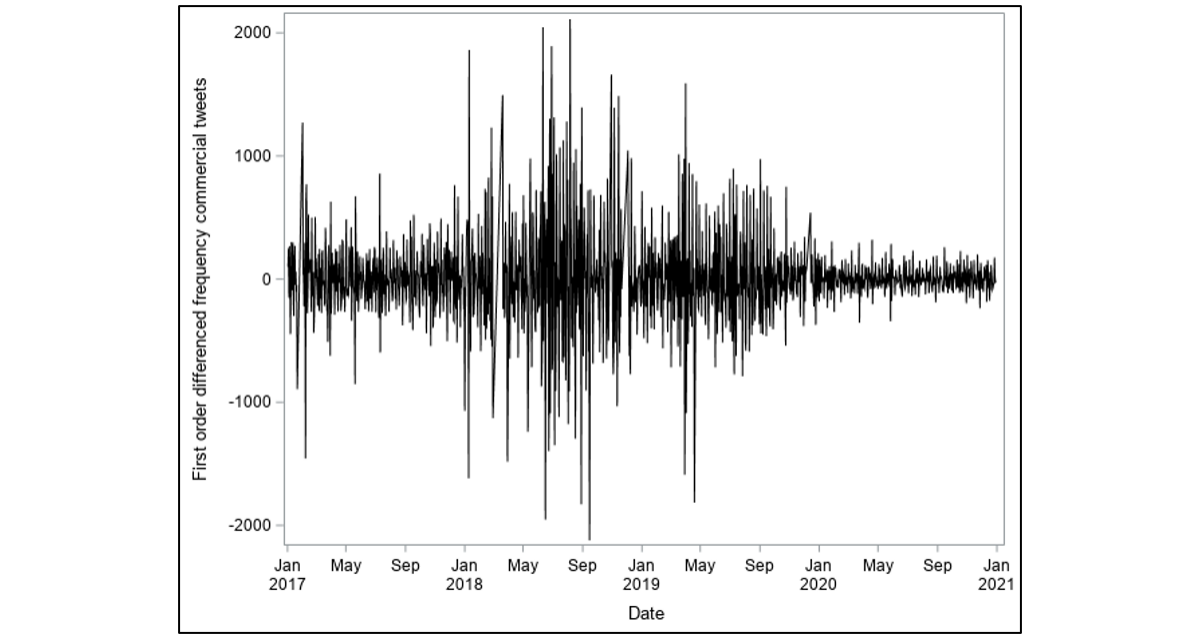
First-order differenced frequency of commercial tweets from January 1, 2017, to December 31, 2020.

**Figure 6:**
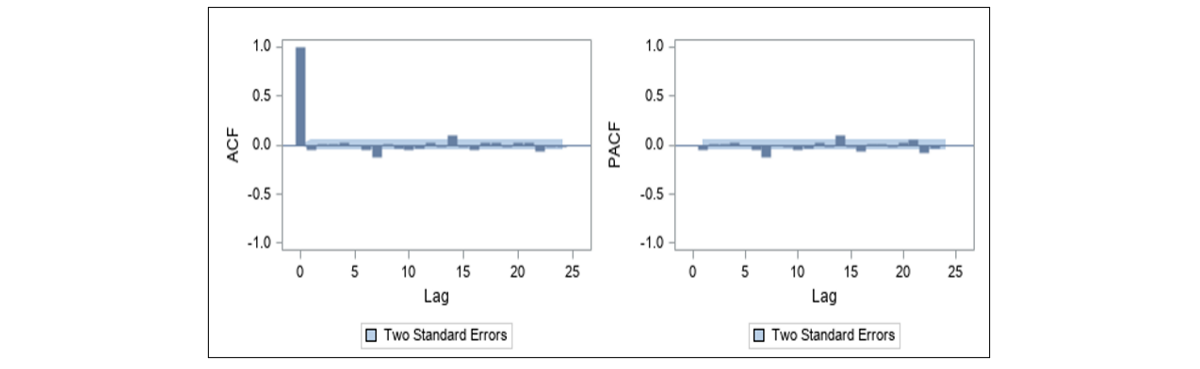
Autocorrelation function (left panel) and partial autocorrelation function (right panel) plots for daily commercial tweets about e-cigarettes after first-order differencing for the autoregressive integrated moving average model. ACF: autocorrelation function; PACF: partial autocorrelation function.

The frequency of daily commercial tweets shown in Figure 1 does not appear to suggest the presence of seasonal or cyclical trends in the data. The identification stage of the data showed that the series is nonstationary, as depicted in the ACF and PACF plots in Figure 4. The ACF plot of a stationary series will decay to zero relatively quickly, which is not the case in Figure 4. We performed a first-order differencing of the series in order to establish stationarity (see Figure 5). The differenced series suggests that AR(7) and MA(1) were appropriate for the data. This suggests that the model uses commercial tweets about e-cigarette for the past 7 days to forecast the frequency of commercial tweets for the next day. The ACF and PACF plots of the final higher order ARIMA model with *p*=7 and *q*=1 are presented in Figure 6. These plots suggest that the fitted model yields a better fit to the data.

#### UCM Approach

**Figure 7:**
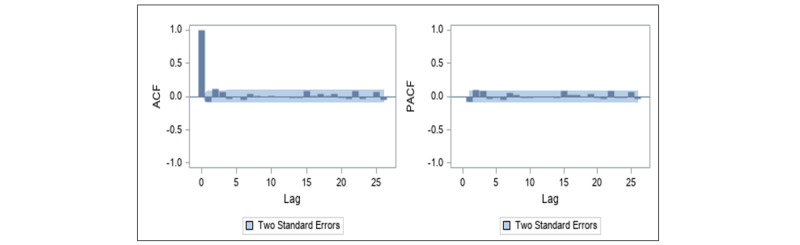
Autocorrelation function (left panel) and partial autocorrelation function (right panel) plots for daily commercial tweets about e-cigarettes for the unobservable components model. ACF: autocorrelation function; PACF: partial autocorrelation function.

The first fitted UCM included only the trend and irregular components. The final estimates of the free parameters for the UCM with only irregular and trend components are presented in Table 2. This table shows the variances of the irregular, slope, and level components. The results suggest fixing the variance of the slope component to zero (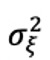
=0.00, *P*=.99) while inferring stochastic irregular (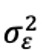
=82530, *P*<.001) and stochastic level (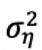
=13043, *P*<.001) components. Subsequent specification of the UCM, by fixing 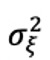 to zero, suggests dropping the slope component from the model (*χ*^2^_1_=0.06, *P*=.81). The final specified UCM, after dropping the slope component, includes irregular and level components and all 4 predictors (ie, FDA events, other events, day, and JUUL). The ACF and PACF plots shown in Figure 7 suggest that the specified UCM with all 4 predictors was a good fit to the data.

**Table 2:** Final estimates of free parameters of the unobservable components model.

Component	Parameter	Estimate	SE	*t*-score	*P* value
Irregular	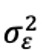	82530	4938.90	16.71	<.001
Level	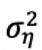	13043	2533.70	5.15	<.001
Slope	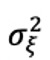	0	0	0.01	.99

#### Model Comparison

Four measures were used to evaluate the predictive performance of the ARIMA model and UCM. The prediction accuracy of the models is summarized in Table 3. The results show that the UCM outperformed the ARIMA model. From Table 3, the MAPE indicates that, on average, the predicted values of the UCM are only off by about 12% compared to 31% for the ARIMA model. Similarly, the UCM produced the smallest RMSE (102.47) estimates, indicating that the UCM is more appropriate for our data. The MAD suggests that the UCM resulted in the smallest MAD (65.08) between the predicted frequency of commercial tweets and the observed frequency of commercial tweets. Finally, the findings show that 84% of the variability in the commercial tweets is well-described components in the UCM compared to 79% when the data were fitted with ARIMA model.

**Table 3:** Fit indices based on residuals for various models.

Criterion	Model	
	ARIMA^a^	UCM^b^
RMSE^c^	314.62	102.47
MAPE^d^ (%)	31.20	11.98
MAD^e^	190.60	65.08
*R^2^^f^*	0.79	0.84

^a^ARIMA: autoregressive integrated moving average.

^b^UCM: unobservable component modeling.

^c^RMSE: root mean squared error.

^d^MAPE: mean absolute percentage error.

^e^MAD: mean absolute deviation.

^f^*R*^2^: coefficient of determination.

#### Predictors of Commercial Tweets About e-Cigarettes

All 4 explanatory variables included in the UCM were significant predictors of the frequency of commercial tweets about e-cigarettes. The results of the predictors are presented in Table 4. The results indicate that, on average, commercial tweets about e-cigarette on the days with FDA events were significantly higher by around 20 tweets per day after accounting for other variables (*β*=19.32, *P*<.001). The coefficient associated with “other” event was 7.74. This implies that commercial tweets about e-cigarette on the days with other major events were significantly higher by around 8 tweets per day, after accounting for other variables, on average (*β*=7.74, *P*=.001). Compared to weekdays, the results show that there were significantly fewer commercial tweets about e-cigarettes on weekends by around 5 tweets after accounting for other variables (*β*=–4.73, *P*=.001). Furthermore, we found that, on average, commercial tweets about e-cigarettes when JUUL’s Twitter account was active were significantly higher by around 171 tweets per day, after accounting for other variables (*β*=170.68, *P*<.001).

**Table 4:** Unobservable components model analysis summary for explanatory variables in the model.

Predictors	Estimate	SE	*t-*score	*P* value
FDA^a^ event	19.32	3.65	5.29	<.001
Other event	7.74	2.35	3.30	.001
JUUL	170.68	38.29	4.46	<.001
Weekend	–4.73	1.48	–3.19	.001

^a^FDA: US Food and Drug Administration.

## Discussion

### Principal Findings

Brand marketing and promotion of e-cigarette products on social media are currently unregulated in the United States. The lack of social media surveillance means that youths are continually exposed to digital marketing of e-cigarette products. As one study reports, Twitter expanded the reach of information about e-cigarettes by 10-fold [49]. Our study contributes to knowledge about factors that drive how commercial companies engage in brand marketing and advertising of e-cigarette products. This analysis used the UCM to model the daily frequency of commercial tweets about e-cigarettes. Previous studies that explored brand marketing and advertising of e-cigarettes only used descriptive statistics to describe the frequency of tweets [11,20,50]. Thus, a strength of this study is the use of 4 explanatory variables to predict the daily frequency of commercial tweets about e-cigarettes. We used data on commercial tweets about e-cigarettes collected over a 4-year period to investigate this.

We found that the daily frequency of commercial tweets was, on average, higher on days with FDA-related events and other non-FDA important events. One possible explanation of this result is that manufacturers of e-cigarette products flood the Twitter space with digital marketing on days with major FDA announcements. For example, there were 3782 commercial tweets about e-cigarettes on September 11, 2018. This was the highest frequency of commercial tweets recorded during an FDA-related event within our data collection period (ie, from January 1, 2017, to December 31, 2020). Remarkably, there were 2 important FDA-related events on this day. First, the FDA issued a statement on “new steps to address epidemic of youth e-cigarette use” [51]. Second, the FDA issued warning letters to more than 1300 retailers and 5 major manufacturers for their roles in perpetuating youth access [52]. There was a noteworthy spike in the number of commercial tweets on the same day that the FDA issued these letters. Research has shown that manufacturers of e-cigarettes use paid social media influencers to promote their products. The spike recorded on September 11, 2018, may suggest that FDA-related events or other major events are a part of marketing plans of e-cigarette manufacturers. In a March 17, 2021, brief, the FDA requested marketing documents from 4 manufacturers of e-cigarette products to understand how these commercial companies engage their users on social media. This analysis provides evidence of trends in brand marketing and advertisement of e-cigarette products when there are important announcements.

In late 2019, some social media platforms restricted paid advertising of tobacco products on their platforms. Twitter’s policy states that “Twitter prohibits the promotion of tobacco products, accessories, and brands globally” [25]. We observed a decline in the frequency of commercial tweets after these social media platforms restricted paid advertising of tobacco products. Interestingly, JUUL stopped tweeting from its corporate account on August 29, 2019, coinciding with the period that some social media companies moved to ban paid advertising of tobacco products on their platforms. We observed that there were, on average, 1000 fewer commercial tweets about e-cigarettes in 2020 compared to the previous years in this study (ie, 2017 to 2019). This demonstrates that tobacco companies still get around these policies through nonpaid advertisements and use of paid social media influencers [21,22].

Adequately modeling our data was essential to provide policymakers with appropriate tools to forecast daily patterns in commercial tweets about e-cigarettes. To find the best-fitting model for our data, we compared the prediction accuracies of 2 statistical models: ARIMA and UCM. The prediction accuracies of the ARIMA model and UCM were judged using MAPE, MAD, RMSE, and *R*^2^ statistics. The results demonstrate the utility of UCM in predicting daily commercial tweets about e-cigarettes. We showed that UCM was an improvement over ARIMA. Unfortunately, forecasting in ARIMA is limited to past behavior of the variable (ie, frequency of commercial tweets). This implies that the effects of other factors or confounding variables cannot be modeled in ARIMA. In addition, outliers are difficult to forecast in ARIMA [45]. The UCM compensates for ARIMA as it provided the luxury to capture different components in the series. In addition, we included 4 explanatory variables in the UCM. All 4 explanatory variables that we examined significantly predicted the daily frequency of commercial tweets about e-cigarettes.

### Limitations

One limitation of this analysis is that commercial content was investigated using Twitter only. Future studies could explore other social media platforms commonly used among young audiences such as Facebook, Snapchat, and YouTube [53]. Another limitation of this study is the limited period of selected tweets for annotation. Tweets between August 23, 2019, and September 25, 2019, were selected for annotation and subsequent training of the classifier. Another limitation is that we did not develop any mechanisms for filtering out suspicious “bot” accounts, which may include newly opened accounts or accounts with zero followers. The public health community has called for increased surveillance of social bots, which are automated accounts relying on sophisticated artificial intelligence to influence discussion, ideas, or products [54,55]. However, a previous study on e-cigarettes revealed that tweets posted by bot accounts were less than 5% since 2012 [56]. For this reason, we did not use bot detection but see this approach as an important step in future research. We acknowledge that the search terms we used to capture Twitter chatter related to e-cigarettes may not have been exhaustive. Some tweets related to e-cigarettes that did not include any of the search terms that we used may have been missed during data collection. Additional search terms from recent research and trending hashtags should be considered in future work.

Research has shown that manufacturers of e-cigarette products use the services of social media influencers to market e-cigarette products. Our study did not distinguish among type of commercial tweet (eg, whether the tweet was from a corporate marketing account or other accounts such as paid social media influencers). In addition, the classifier developed for this study did not include specific marketing themes of commercial tweets (eg, flavors or price promotions). These could serve as areas of consideration for future studies, especially with the FDA seeking to understand the social media advertising and marketing plans of manufacturers of e-cigarette products. Despite these limitations, the UCM is promising in modeling predictors of commercial tweets about e-cigarettes.

### Conclusion

The aim of this study was to investigate factors that predict changes in daily frequency of commercial tweets about e-cigarettes using time series modeling techniques. Data collected were fitted using 2 time series models, ARIMA and UCM. The results of the UCM, which proved to be the best fitting model, showed that brand advertisement and marketing of e-cigarettes on Twitter was significantly higher on days with FDA-related events compared to days without FDA events after accounting for other variables. In addition, we found higher marketing of e-cigarette products on days with important national news like state legislative actions controlling the use of e-cigarette products and significant scientific research studies. We conclude that e-cigarette companies may increase brand marketing of their products on days with important FDA announcements related to e-cigarettes and days with important national news about e-cigarettes, possibly to alter the narrative about the information shared by the FDA or other important news reporting on e-cigarettes. Our results also reveal significantly higher marketing of e-cigarette products on weekdays compared to weekends. Previous work showed that the use of e-cigarette products decreased during weekends [57]. This leads us to believe that e-cigarette companies, more likely than not, target their audience the most during weekdays.

## Abbreviations

ACF: autocorrelation function
AR: autoregressive parameter
ARIMA: autoregressive integrated moving average
AUROC: area under the receiver operating characteristic
BERT: bidirectional encoder representations from transformers
CASAA: Consumer Advocates for Smoke-Free Alternatives Association
CNN: convolutional neural network
e-Cigarette: electronic cigarette
FDA: US Food and Drug Administration
LSTM: long short-term memory
MA: moving average
MAD: mean absolute deviation
MAPE: mean absolute percentage error
PACF: partial autocorrelation function
*R* ^2^: coefficient of determination
RITHM: real-time infoveillance of Twitter health messages
RMSE: root mean square error
UCM: unobservable components model
